# Supplementary figures and images for: An arginine switch drives the stepwise activation of β-arrestin by CXCR7
Source: PLoS Biol. 2025 Aug 7;23(8):e3003312. doi: 10.1371/journal.pbio.3003312 (PMC12331092; doi:10.1371/journal.pbio.3003312)

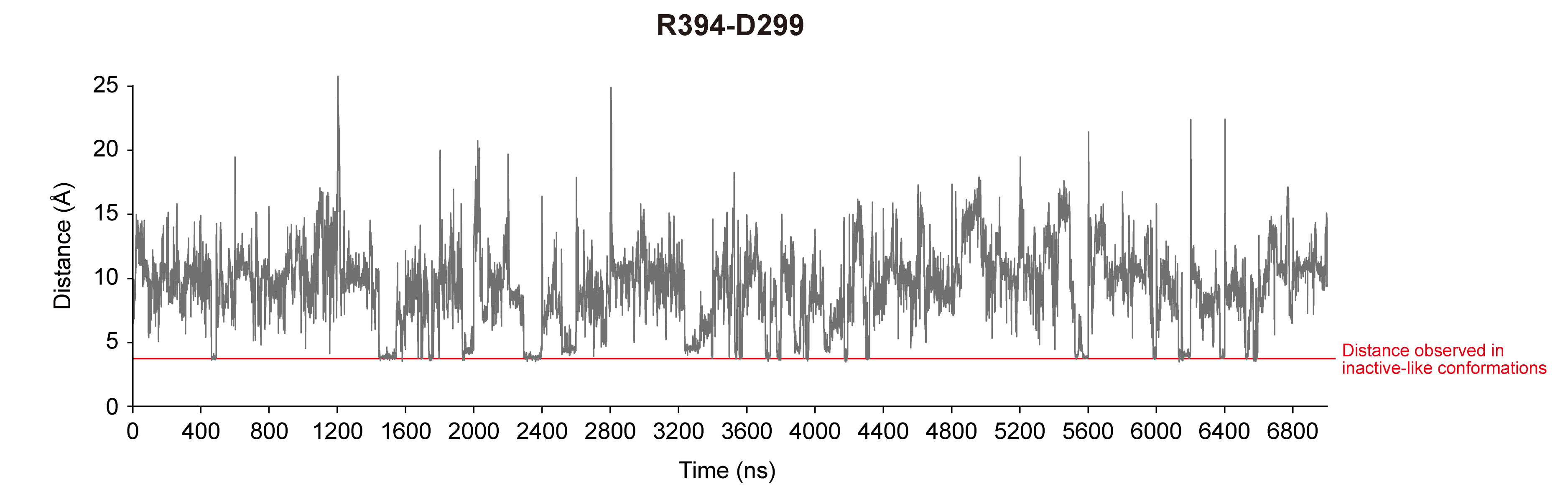

Supplement: S1 Fig — The distance between R394 and D299 (forming polar interactions in the inactive state of βarr2) monitored within MD simulations (35 × 200 ns). The data underlying the graphs shown in the figure can be found in S1 Data. (TIF) [file pbio.3003312.s001.tif]

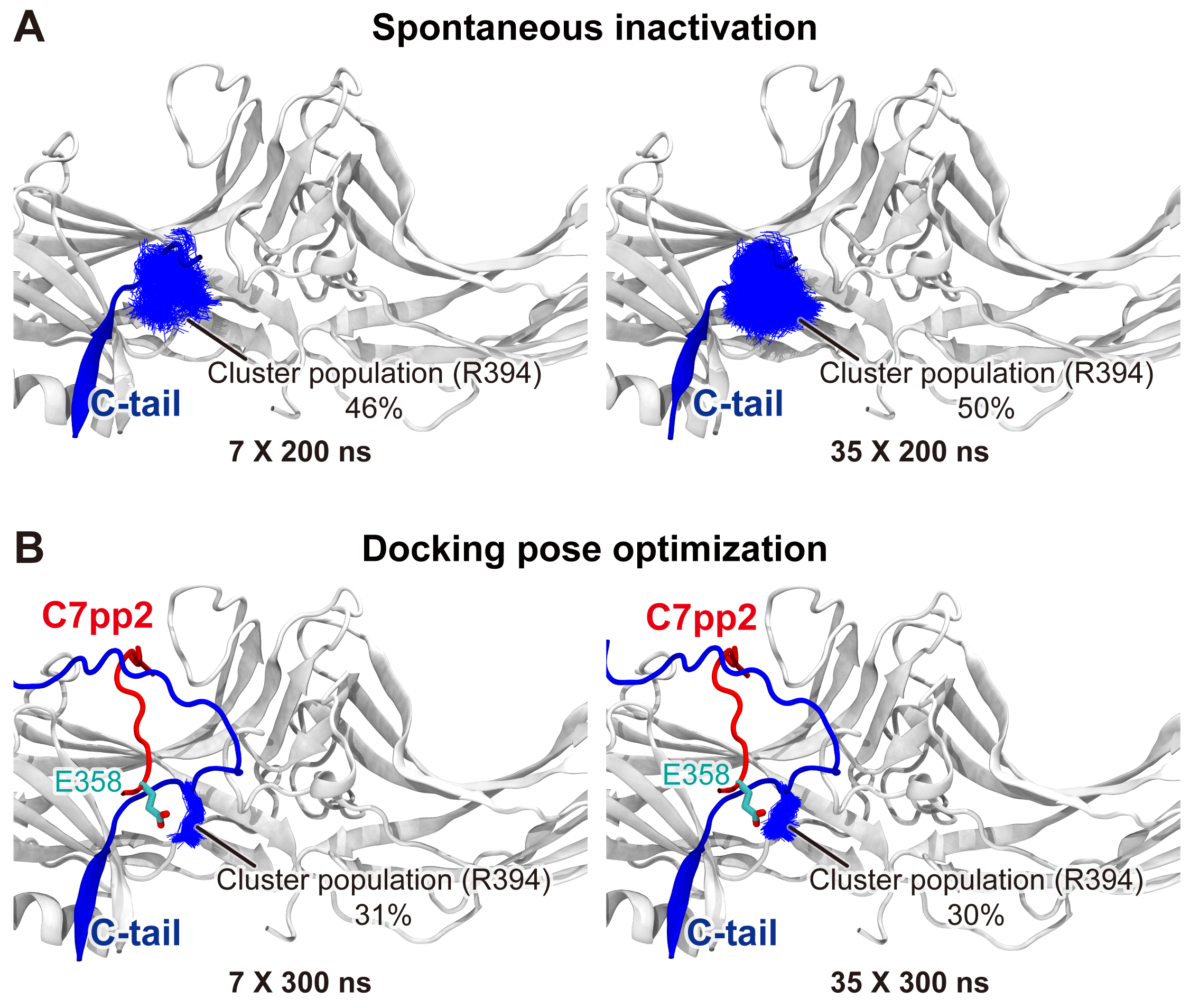

Supplement: S2 Fig — (A, B) Main cluster conformations of R394 during spontaneous inactivation (A) and Docking pose optimization (B) are shown in blue sticks. The population of each R394 cluster is indicated. (TIF) [file pbio.3003312.s002.tif]

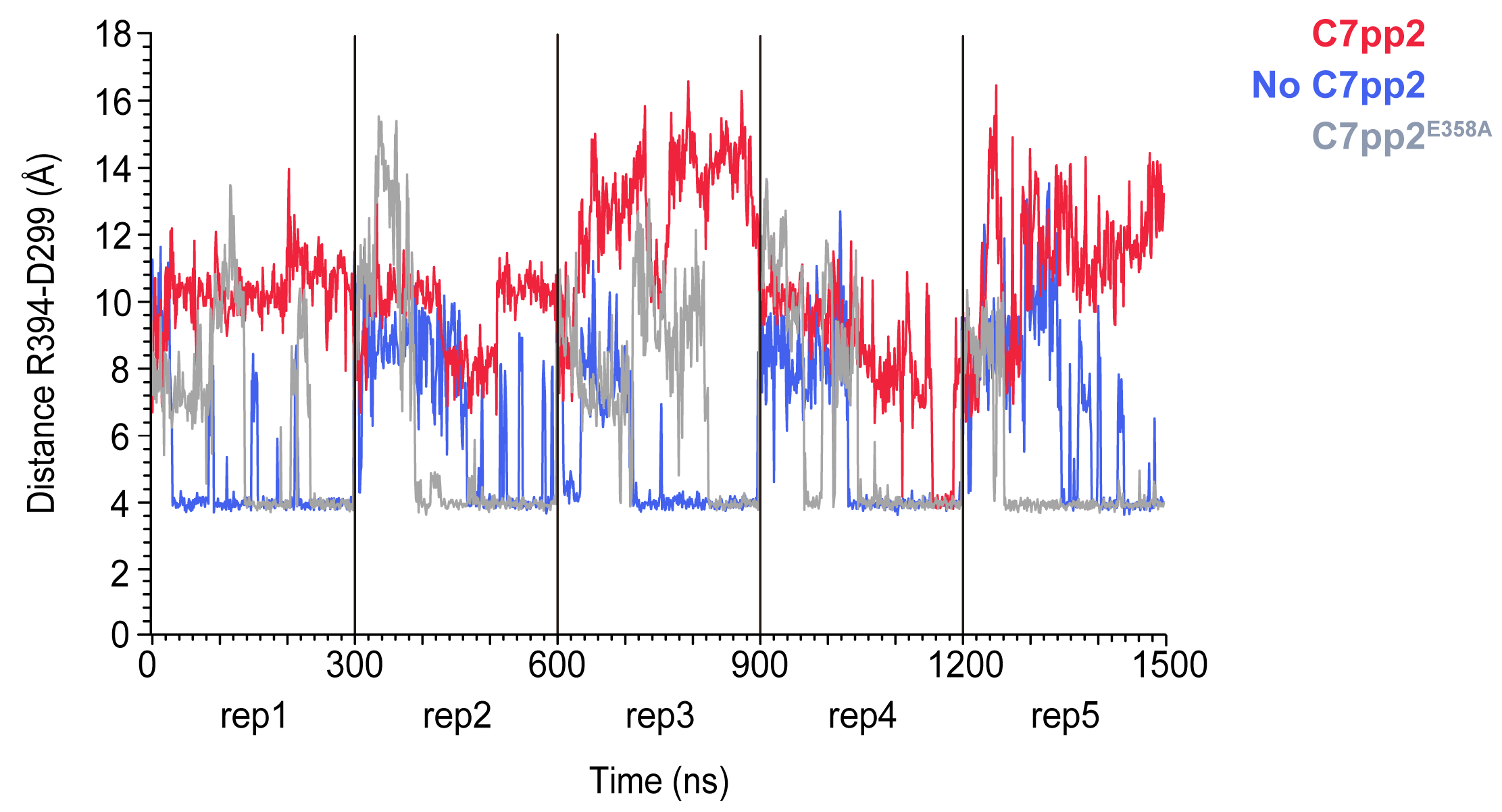

Supplement: S3 Fig — The data underlying the graphs shown in the figure can be found in S1 Data. (TIF) [file pbio.3003312.s003.tif]

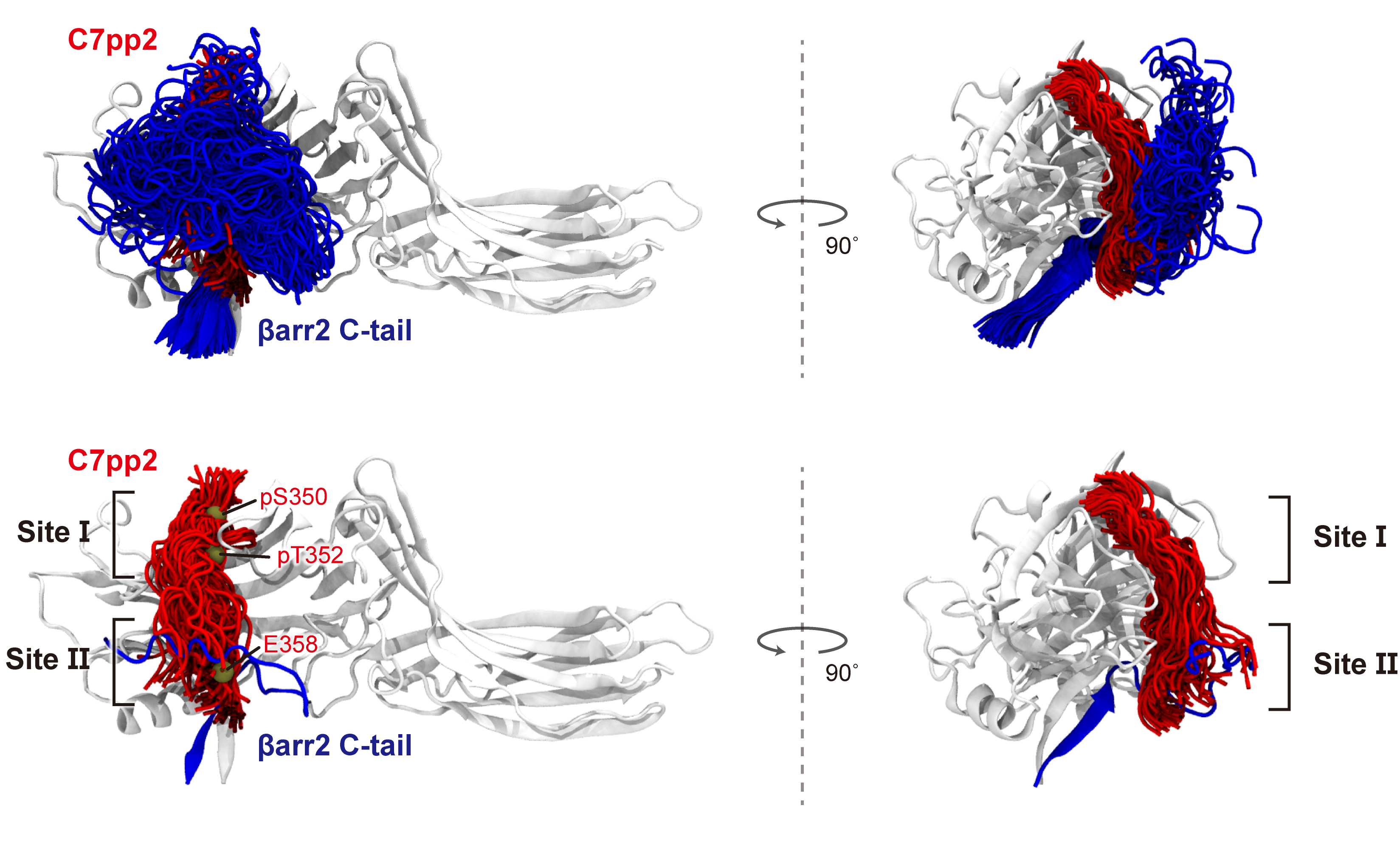

Supplement: S4 Fig — (TIF) [file pbio.3003312.s004.tif]

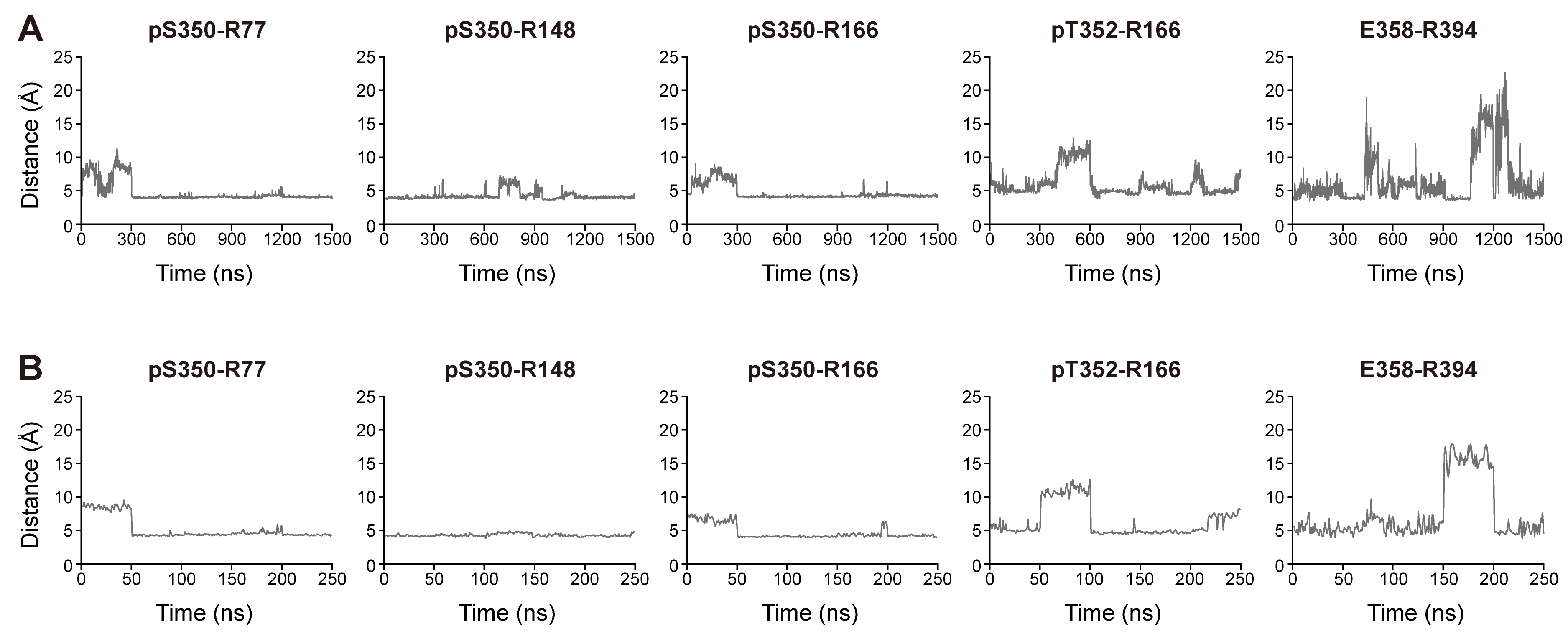

Supplement: S5 Fig — (A, B) The distance between residues in site I (pS350 and pT352 of C7pp2; R77, R148, and R166 of βarr2) and site II (E358 of C7pp2 and R394 of βarr2) are monitored during the whole simulation run (panel A, 5 × 300 ns) and the last 50 ns of each simulation replicate (panel B, 5 × 50 ns). For site I, the distance between the phosphorus (P) atom of the phosphorylated residues (pS350 and pT352) and the carbon atom in the guanidinium group (Cζ) of the corresponding arginine residues (R77, R148, and R166) are plotted. For site II, the distance between the Cδ atom of E358 and the Cζ atom of R394 is plotted. The data underlying the graphs shown in the figure can be found in S1 Data. (TIF) [file pbio.3003312.s005.tif]

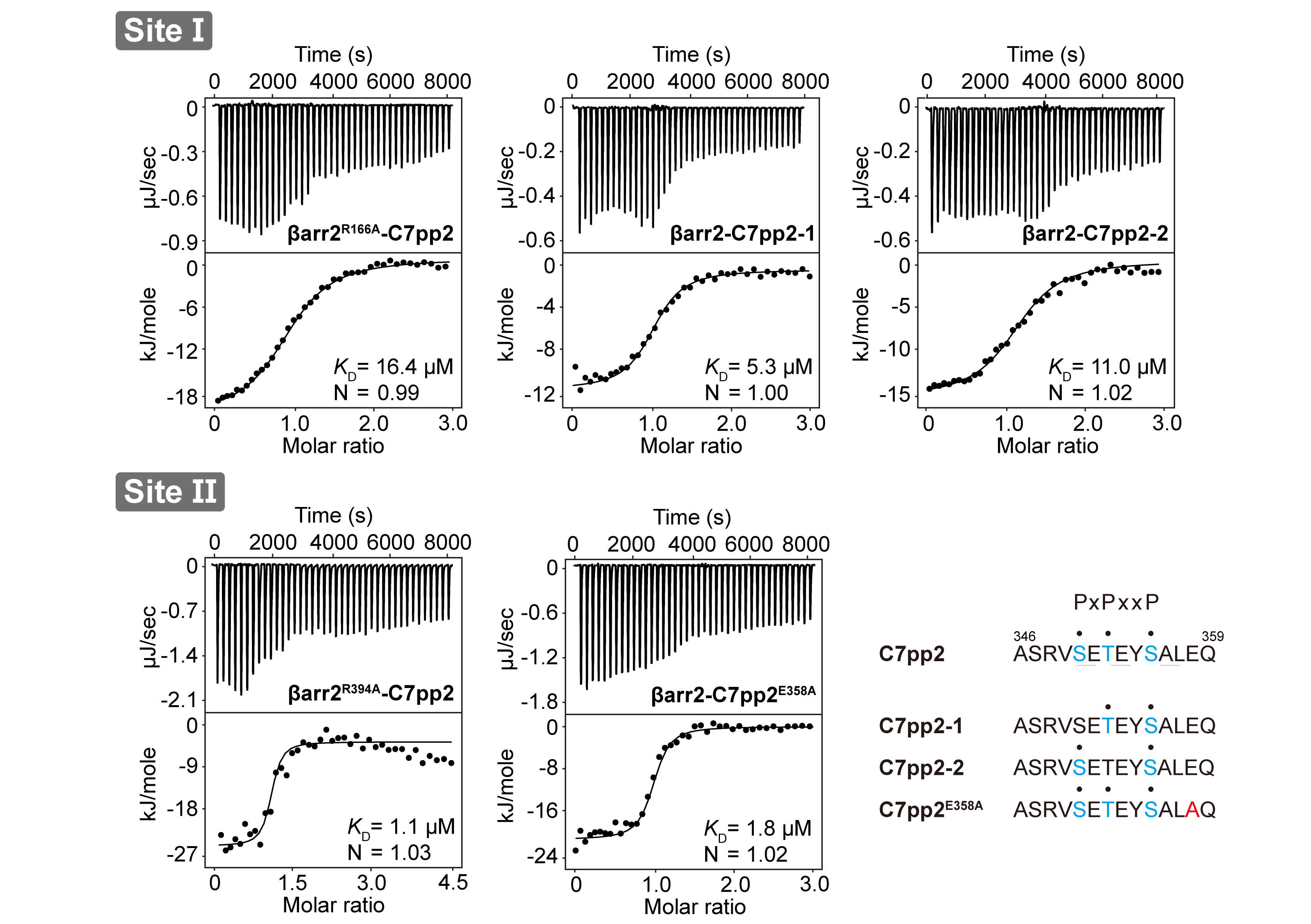

Supplement: S6 Fig — ITC experiments showing the effect of mutations in the binding interfaces between βarr2 and C7pp2. The detailed sequences of C7pp2 mutants used for the ITC assay are shown in the lower right panel. Purified βarr2 was incubated with increasing peptide concentrations, and the binding parameters were calculated based on the dose-response curve. The data underlying the graphs shown in the figure can be found in S1 Data. (TIF) [file pbio.3003312.s006.tif]

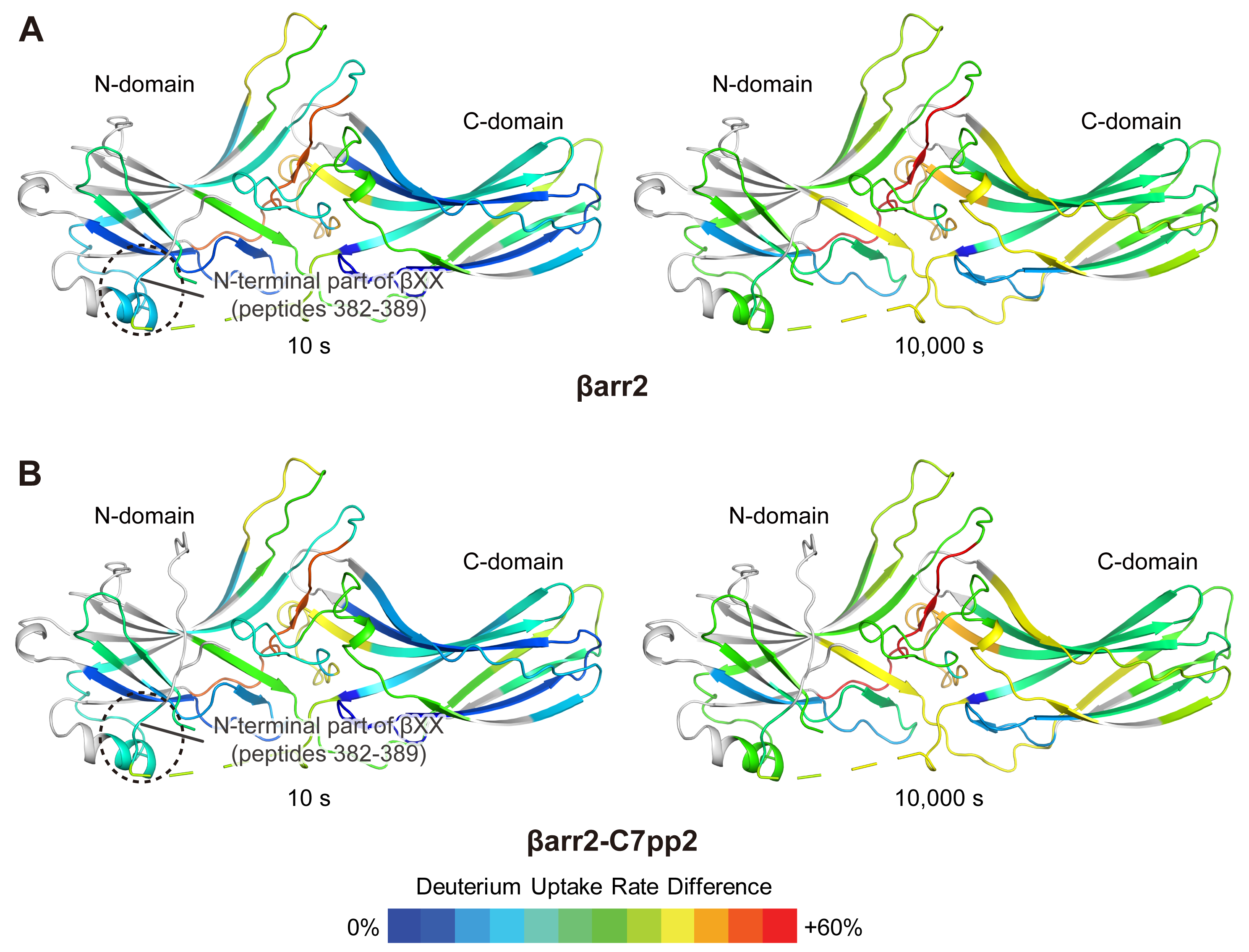

Supplement: S7 Fig — (A, B) The deuterium uptake profiles were mapped onto the structure of βarr2 (panel A) and βarr2-C7pp2 (panel B). Deuterium incorporation after 10 and 10,000 seconds of D2O buffer incubation is indicated by a color code. The color legend represents the level of deuterium uptake, and uncovered regions are shown in gray. N-terminal part of βXX region in the structure is highlighted as black dotted circle. (TIF) [file pbio.3003312.s007.tif]

**Fig 1B**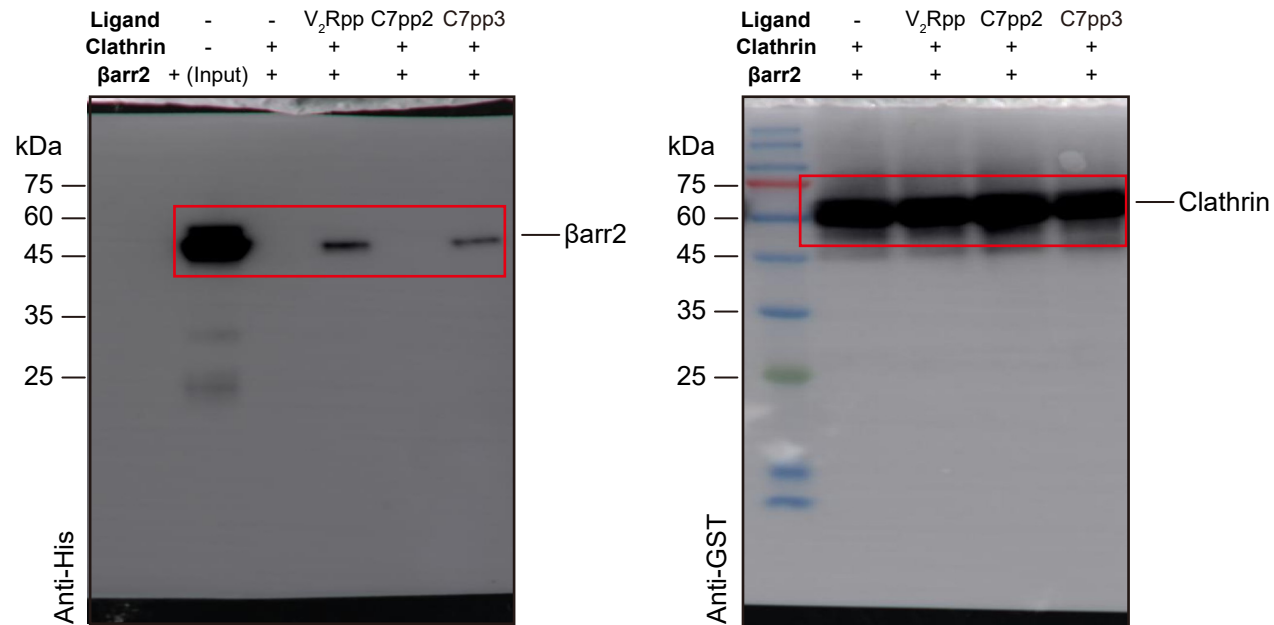

Supplement: S1 Raw Images — (PDF) [file pbio.3003312.s010.pdf]
