# Supplementary material for: An arginine switch drives the stepwise activation of β-arrestin by CXCR7
Source: PLoS Biol. 2025 Aug 7;23(8):e3003312. doi: 10.1371/journal.pbio.3003312 (PMC12331092; doi:10.1371/journal.pbio.3003312)
Supplement: Attachment — Submitted filename: Authors_ response to reviewers_ comments_20250415_002.docx [file pbio.3003312.s013.docx]

**Authors’ Response to Reviewers’ Comments**

**Reviewer #1 (Remarks to the Author):**

This manuscript describes an intermediate state in the activation of barr2 through binding the phosphorylated C-terminal tail of a GPCR using molecular dynamics simulations, ITC, a cell-based assay, and HDX-MS. Specifically, the authors are interested in examining how the sequestered barr2 C-terminal tail, which binds the N-domain and stabilizes the inactive state, is released upon interaction with GPCRs. Previous structural data has been limited to the active and inactive states of barr, leaving the mechanisms occurring in the intermediate states unknown. Using MD, the authors find that a peptide representing a phosphorylated GPCR C-terminal tail binds to barr2 while the barr2 C-tail is still bound to the N-domain in an intermediate state, identifying a previously unrecognized binding mode. The manuscript is well-written and appears overall technically sound. I have the following suggestions to improve the presentation and interpretability of the data:

***RESPONSE***

We sincerely appreciate the reviewer’s time and effort in carefully reviewing our manuscript and providing valuable feedback to improve it. The reviewer’s insightful comments and constructive suggestions have been highly beneficial in refining our manuscript, enhancing the clarity and interpretability of our findings. We have carefully considered all of the reviewer’s suggestions and have incorporated the necessary revisions to ensure that our study is communicated more effectively.

The following paragraphs outline the specific revisions made in response to the reviewer’s suggestions and explain how they have been integrated into the revised manuscript. To ensure clarity and prevent any potential misunderstandings arising from discrepancies in line numbers, all revised sentences in the response are enclosed in double quotation marks, with modified or newly inserted text specifically highlighted in blue for emphasis.

We hope that these changes contribute to a more comprehensive and accessible presentation of our findings and that our responses adequately address the reviewer’s valuable suggestions.

**Main points**

[Reviewer #1- main point 1]
I recommend including a domain layout of barr2 with the domain and structural features discussed in the manuscript highlighted.

***RESPONSE***

We sincerely appreciate the reviewer’s thoughtful suggestion to include a domain layout of βarr2 highlighting structural features discussed in the manuscript. We fully agree that providing such a representation will enhance clarity and help readers better understand the key structural elements relevant to our findings.

In response to this suggestion, we have revised Figure 1 to include additional annotations highlighting the key βarr2 domains and structural features discussed throughout the manuscript (including the N-domain, C-domain, C-tail, polar core region, β-strand XX, and gate loop). We believe that this modification will improve the accessibility of our study.

Fig 1C. Ribbon diagram of inactive βarr2. The C-tail of inactive βarr2 is highlighted in red. The polar core region, gate loop, and βXX motif are indicated. A zoomed-in view of the polar core region (right panel) shows an initial step of the simulation. The C-tail of βarr2 (residues 385 to 397) was manually displaced (shown in green) from the initial inactive conformation. The observed spontaneous inactivation of the C-tail during the simulation is indicated by a dotted arrow.

[Reviewer #1- main point 2]

Line 106-107, how do the authors know that C7pp2 is binding and inducing an intermediate state? This seems like a jump in rationale.

***RESPONSE***

We appreciate the reviewer’s comment regarding the logical flow of our manuscript. We agree that this statement could potentially cause confusion for readers of the manuscript.

It is indeed true that we hypothesized the induction of an intermediate state upon C7pp2 binding for the following reasons. As described in lines 90-93, previous work demonstrated that the phosphorylation-site cluster 2 in CXCR7, which includes the sequence corresponding to C7pp2, is critical for βarr2 trafficking [1]. Mutation of the cluster 2 sites in CXCR7 significantly impaired agonist-induced βarr2 recruitment and subsequent endosomal localization [1]. Moreover, in this work, the clathrin binding assay results indicated that the phosphorylation of C7pp2 at three sites is insufficient to fully displace the βarr2 C-tail. By combining these two findings, we anticipated that MD simulations with C7pp2 could reveal a βarr2 conformation distinct from the previously characterized fully active state, providing new insights into the activation process.

However, we agree with the reviewer’s comment that our manuscript, as currently written, does not clearly describe this logical flow and thus could be interpreted as a logical leap. Therefore, to enhance interpretability, we have revised the manuscript as follows.

Lines 129-131:

“To better understand the interaction dynamics where the βarr2 C-tail is not fully displaced when bound to C7pp2, we used a sequential modeling protocol of molecular dynamics simulations.”

[Reviewer #1- main point 3]

In figure 1C, I would recommend not coloring the aspartate sidechains red or coloring the oxygens a different shade of red.

***RESPONSE***

We appreciate the reviewer’s suggestion and have modified the color of the side chains accordingly to better distinguish them from the oxygen atoms. Additionally, we have revised all structural figures throughout the manuscript to ensure clear differentiation of side-chain atoms.

[Reviewer #1- main point 4]

In the legend for figure 3, please include information on the statistical analysis that was performed and significance.

***RESPONSE***

We appreciate the reviewer’s thoughtful suggestion and fully agree that including details on statistical analysis and significance is essential for clarity. In response, we have revised the legend for Figure 3 to incorporate the necessary statistical analysis and significance for all panels. The revised figure now clearly specifies the statistical tests performed and the significance levels to ensure transparency and better interpretation of the data.

Fig 3. Destabilization of the polar core by C7pp2. (A) Comparison of polar core and three-element interactions between the inactive and intermediate conformations of βarr2. The ribbon diagram of βarr2 is colored dark gray. C7pp2 and the C-tail of βarr2_IM_ are shown in pink and green, respectively. Hydrogen bonds stabilizing the polar core interactions are shown as dashed lines. (B) Boxplots comparing the distance between D299 and R394 (forming polar interactions in the inactive βarr2) monitored within MD simulations (5 × 300 ns). Outliers are depicted as points. The significance of differences between the groups was measured using a two-sided Mann–Whitney U test. (C) CXCL12-induced trafficking of βarr2 as monitored using confocal microscopy in HEK293 cells expressing CXCR7^WT^ and CXCR7^E358A^. The "Surface" images depict βarr2 localization at the plasma membrane shortly after agonist stimulation, representing initial recruitment of βarr2 to CXCR7. The "Endosomal" images show intracellular βarr2 localization at later time points, indicating receptor internalization. Scale bar is 10 μm. (D) The whole-cell ELISA-based assay to measure the surface expression of CXCR7. Data are presented as mean ± SEM of three independent experiments, normalized to CXCR7^WT^. (E) CXCL12-induced βarr2 recruitment to CXCR7^WT^ and CXCR7^E358A^ in the Tango assay. Data are presented as mean ± SEM. CXCR7^E358A^ is significantly compromised in inducing βarr2 trafficking upon CXCL12 stimulation.

[Reviewer #1- main point 5]

I suggest moving figure S5 into the main text. I find it to be much more informative and clear with regards to following the text than current figure 3A. If 3A was replaced and 3B made smaller, I think S5 would fit within 3.

***RESPONSE***

We appreciate the reviewer’s insightful suggestion. As recommended, we have moved Figure S5 into the main figure, replacing Figure 3A, and adjusted Figure 3B accordingly. This revision has indeed made the figure more informative and improved the overall clarity of the manuscript. We sincerely thank the reviewer for this valuable recommendation, which has enhanced the presentation of our findings.

[Reviewer #1- main point 6]

In figure 3C, what does "surface" and "endosomal" refer to? How are these treatments different? This information should be included either in the figure legend or the results section. Some description of the assays used in figure 3C-E should be included in the results section (lines 212-216) to help the reader understand why these assays were used.

***RESPONSE***

We appreciate the reviewer’s insightful suggestion. We agree that the distinction between "surface" and "endosomal" may not have been immediately clear, and other readers might also find this aspect ambiguous. Given this, we believe it is important to incorporate the reviewer’s recommendation to enhance clarity.

To address this, we have revised the legend for Figure 3C to explicitly define what surface and endosomal refer to.

Lines 232-235:

“The "Surface" images depict βarr2 localization at the plasma membrane shortly after agonist stimulation, representing initial recruitment of βarr2 to CXCR7. The "Endosomal" images show intracellular βarr2 localization at later time points, indicating receptor internalization.”

Additionally, we have expanded the Results section by providing a more detailed explanation of the assays used in Figure 3C-E, clarifying their purpose and significance in the context of our study.

Lines 266-271:

“Moreover, confocal microscopy revealed that, unlike CXCR7^WT^, the CXCR7^E358A^ mutant failed to induce βarr2 redistribution from the plasma membrane to intracellular endosomes upon CXCL12 stimulation, suggesting impaired receptor internalization (Fig 3C). Additionally, the Tango assay demonstrated that CXCR7^E358A^ exhibited a significant reduction in βarr2 recruitment in response to CXCL12, confirming that the E358A mutation disrupts βarr2 engagement and downstream trafficking (Fig 3E).”

We appreciate the reviewer’s suggestion, which has helped improve the manuscript’s clarity and readability.

[Reviewer #1- main point 7-1]

The results section describing the HDX-MS data needs further explanation. Why could data not be obtained on the distal C-tail region? This could signify that something is wrong with the sample, unless further information indicating otherwise is provided. Is the 382-389 region the only part of this tail that data could be obtained on?

***RESPONSE***

We appreciate the reviewer’s concern and the opportunity to clarify this point. HDX-MS data rely on successful peptide identification via mass spectrometry. Factors such as the chemical properties of specific peptides, their ionizability, and the sensitivity of the mass spectrometer and LC system influence peptide identification. Based on our extensive experience with HDX-MS analyses of βarrs [2–11], we have consistently observed that peptides from the distal C-terminus of βarrs are challenging to identify. This is primarily due to the intrinsic chemical nature of this region rather than from any issues with sample quality.

Accordingly, in the current study, peptide 382–389 is the only segment within this region (spanning βXX) for which HDX-MS data are available.

[Reviewer #1- main point 7-2]

I would recommend that the authors also show the complete HDX-MS data showing the overall dynamics of barr2 and the peptide plotted onto each structure (alone and in complex, either one chosen time point or an average), for completeness and show this in the supplemental. Per peptide deuterium uptake levels are shown in an Excel file in the supplemental, but it is not easy for the reader to interpret the overall dynamics of barr2 by looking at this.

***RESPONSE***

Thank you for this suggestion. As noted, we have extensively studied the HDX-MS profiles of βarrs in previous studies. Particularly, the overall dynamics of βarr2 have been reported in the previous publication [2].

In the current manuscript, we chose to provide per-peptide deuterium uptake levels in an Excel file in accordance with the HDX-MS reporting standards outlined in the literature [12]. These recommendations ensure transparency and reproducibility in HDX-MS data presentation.

However, we understand the importance of facilitating data interpretation for readers. Therefore, we include supplementary figures mapping the HDX-MS data onto the structure of βarr2 (both alone and in complex with C7pp2) at 10- and 10,000-seconds time points (S7 Fig). This figure will provide a visual representation of the overall dynamics and complement the Excel file for clarity.

**S7 Fig. Comparison of HDX-MS data of βarr2 alone and βarr2-C7pp2.** (A and B) The deuterium uptake profiles were mapped onto the structure of βarr2 (panel A) and βarr2-C7pp2 (panel B). Deuterium incorporation after 10 and 10,000 seconds of D_2_O buffer incubation is indicated by a color code. The color legend represents the level of deuterium uptake, and uncovered regions are shown in grey. N-terminal part of βXX region in the structure is highlighted as black dotted circle.

[Reviewer #1- main point 7-3]

Also, what is the relevance of the decreased exchange rates in the middle loop, lariat loop, and back loop? These features are not discussed elsewhere in the text. Please include a discussion of this.

***RESPONSE***

Thank you for pointing out the need for additional discussion on the interpretation of the HDX results. We have incorporated the following description into the manuscript to clarify the new insights derived from our HDX analysis

Lines 292-293:

“These observations raise the possibility that binding of C7pp2 to βarr2 could induce conformational rearrangements throughout the βarr2 domains.”

[Reviewer #1- minor point 8]

I like the mechanistic model in figure 5 - well done!

***RESPONSE***

We sincerely thank the reviewer once again for the positive assessment of our manuscript and for providing valuable suggestions to further improve our work.

**Reviewer #2 (Remarks to the Author):**

In the manuscript entitled "An arginine switch drives the stepwise activation of β-arrestin" by Jeong Seok Ji, et al. the authors use molecular dynamics, ITC binding assays, fluorescence microscopy, and HDX experiments to describe an interaction between β-arrestin and a phosphopeptide of ACKR3 that they claim is important for the activation of β-arrestins. The paper does a good job of characterizing this interaction and providing multiple sources to support the importance of a conserved arginine residue (R394) and its role in destabilizing the polar core and subsequent releasing of the C-terminal tail to allow for β-arrestin activation. While the authors describe a mechanism for the binding of the ACKR3 C-terminal peptide to β-arrestin2 (namely a glutamate residue in the C-terminus of ACKR3 that they propose interacts with R394 of β-arrestin2) they fall short in relating this broadly to GPCRs at large. While the introduction and discussion sections are framed in terms of general mechanisms for arrestin activation by GPCRs, no analysis is done to assess the presence of comparable residues or motifs in other GPCR C-terminal sequences. This omission must be addressed to justify any discussion of general GPCR/arrestin activation mechanisms.

***RESPONSE***

We sincerely appreciate the reviewer’s time and effort in carefully reviewing our manuscript and providing thoughtful feedback. We are grateful for the reviewer’s positive assessment of our study and for recognizing our efforts in characterizing the interaction between βarr2 and the ACKR3 C-terminal phosphopeptide.

We have carefully considered the reviewer’s concerns and have revised the manuscript to prevent any potential misunderstandings regarding the broader relevance of our findings. Additionally, we have thoroughly evaluated and incorporated the reviewer’s suggestions to ensure a more comprehensive and accurate discussion of GPCR-mediated β-arrestin activation. We sincerely appreciate the reviewer’s insightful comments, which have helped strengthen our manuscript.

To ensure clarity and prevent any potential misunderstandings arising from discrepancies in line numbers, all revised sentences in the response are enclosed in double quotation marks, with modified or newly inserted text specifically highlighted in blue for emphasis. We hope that these changes contribute to a more comprehensive and accessible presentation of our findings and that our responses satisfactorily address the reviewer’s valuable suggestions.

**Main points**

[Reviewer #2- main point 1]

What is V2Rpp? It is never described, and its relevance to the ACKR3 peptides that are the focus of the study create confusion at the outset that is never resolved.

***RESPONSE***

We fully agree that the role of V_2_Rpp in our assays was not clearly described, which could cause confusion for readers. To address this, we have revised the manuscript to explicitly explain why V_2_Rpp was used in these assays and its relevance to our study. Specifically, we have added the following sentence to the manuscript.

Lines 99-102:

“Our results showed that C7pp2 binding to βarr2 does not enhance clathrin binding compared to V_2_Rpp, a well-characterized phosphopeptide derived from the C-terminus of the vasopressin type 2 receptor, which effectively leads to the release of βarr2 C-tail (Fig 1B).”

We believe this clarification enhances the manuscript’s readability and ensures that the significance of V_2_Rpp is clearly conveyed. We sincerely thank the reviewer for highlighting this important point.

[Reviewer #2- main point 2]

Lines 33-35 "Our study uncovers a previously unknown molecular switch that, upon engagement, destabilizes the polar core as a crucial step in the GPCR-induced βarr activation.": While the findings in this paper do expand on the importance of a conserved arginine residue in β-arrestins, I would hardly say this was a previously unknown molecular switch. The authors themselves stated "Our analysis specifically focused on Arg394 within the distal C-tail, as previous experimental findings have underscored the conformational rearrangement of this residue as an important step in C-tail displacement50".

***RESPONSE***

We appreciate the reviewer’s thoughtful feedback and fully agree with the concern raised. As we stated in the manuscript, Arg394 has previously been recognized as playing a crucial role in β-arrestin2 activation, therefore, the original statement in the abstract required revision to avoid overstating the novelty of our findings. To address this, we have modified the sentence as follows.

Lines 34-35:

“Our study uncovers a role for the arginine switch that, upon engagement, destabilizes the polar core as a crucial step in the CXCR7-induced βarr activation.”

This revision clarifies that, rather than identifying a completely unknown molecular switch, our study highlights a new functional role of Arg394 specifically in the context of CXCR7-induced β-arrestin activation, which had not been previously described. We have also added 'CXCR7-induced' to emphasize that the proposed mechanism is specific to this receptor context. We thank the reviewer for pointing this out, as this refinement ensures that our findings are communicated more accurately.

[Reviewer #2- main point 3]

Figure 3B - The data shown on the boxplot is inconsistent. All conditions should have replicate points shown as with the first condition (red box). Additionally, some significance analysis could be done between these conditions to convince the reader that these differences are meaningful as the replicates from the WT conditions seem to span the entire range, albeit slightly skewed to the upper distances.

***RESPONSE***

We thank the reviewer for highlighting this issue. The points shown in the first plot correspond to outliers rather than all data points. Thus, the lack of dots in the blue and gray boxplots indicates that no outliers were present in those groups. Following the reviewer’s suggestion, we have included an analysis of the significance of differences between the studied conditions. This has been clarified in the figure legend as follows:

**Fig 3B.** Boxplots comparing the distance between D299 and R394 (forming polar interactions in the inactive βarr2) monitored within MD simulations (5 × 300 ns). Outliers are depicted as points. The significance of differences between groups was assessed using a two-sided Mann–Whitney U test.

[Reviewer #2- main point 4]

Page 22, Line 290 "While our experiments elucidate the activation mechanism of βarr2 by the receptor Rp-tail": The paper describes a potential activation mechanism for the ACKR3 C-terminal peptide and β-arrestin2. This does not necessarily apply broadly to other GPCRs and further analysis of other receptor sequences and potential simulations would have to be done to make this claim. ACKR3 is intrinsically biased toward arrestin, and the authors offer no evidence that this interaction isn't unique to ACKR3. I don't particularly think this is unique to ACKR3 as only the C-terminus was used and it's not particularly different from other GPCRs, but the authors don't even mention this potential pitfall, and this leaves the central claim with some doubt.

***RESPONSE***

We sincerely appreciate the reviewer’s thoughtful comment and fully agree with the concern raised. We also acknowledge that our study cannot be broadly applied to all GPCRs, as our findings are specifically based on CXCR7 and it is important to clarify the scope of our conclusions and prevent any misinterpretation by readers. To address this, we have revised the Discussion section to explicitly state this limitation at the beginning of the section discussing the study's constraints and future directions. The modified text now reads as follows.

Lines 369-373:

“Firstly, this study focuses on CXCR7, and whether the proposed activation mechanism applies to other GPCRs remains to be determined. Although its C-terminal tail shares similarities with other receptors, broader structural comparisons and validation using diverse GPCR phosphopeptides and MD simulations are needed to evaluate the mechanism’s general relevance.”

Additionally, we have revised other parts of the manuscript to ensure that our study's focus on CXCR7 is clearly communicated. This includes modifying certain terms and phrases to prevent overgeneralization and, importantly, revising the title of the manuscript so that readers are aware of this specificity from the outset.

Revised title:

“An arginine switch drives the stepwise activation of β-arrestin by CXCR7”

We are grateful for the reviewer’s valuable feedback, which has helped us refine our manuscript to ensure that our findings are accurately represented and clearly communicated.

**Minor points**

[Reviewer #2- minor point 1]

Page 11, Lines 29-30 "we reveal how specific interactions with a prototypical GPCR": ACKR3 is not a "prototypical" GPCR. It doesn't couple to G-proteins and is intrinsically arrestin biased. The A stands for atypical.

***RESPONSE***

We sincerely appreciate the reviewer’s insightful comment and fully agree with the concern raised. ACKR3 (CXCR7) is not a "prototypical" GPCR, as it does not couple to G-proteins and is intrinsically biased toward arrestin. Given this, the use of "prototypical GPCR" was inaccurate, and we recognize the importance of using terminology that accurately reflects the nature of our study. To address this, we have revised the sentence to explicitly reference CXCR7, the actual focus of our study, rather than using the "prototypical GPCR" descriptor.

Lines 30-31:

"we reveal how specific interactions with a chemokine receptor 7 (CXCR7) promote the unbinding of the βarr2 C-tail—a crucial step in arrestin activation.”

[Reviewer #2- minor point 2]

Page 12, Lines 44-46 "The removal of GPCRs from the cell surface through βarr-mediated internalization prevents them from transmitting extracellular signals into cells.": This sentence is followed by a contradictory statement and should be revised to clarify and harmonize the intended meaning (e.g. "continued transmission of extracellular")

***RESPONSE***

We sincerely appreciate the reviewer’s suggestion. To improve the logical flow of the Introduction and enhance clarity, we have revised the sentence as follows.

Lines 45-46:

“The removal of GPCRs from the cell surface through βarr-mediated internalization limits the continued transmission of extracellular signals into the cell. However, …”

[Reviewer #2- minor point 3]

Page 16, Lines 151-154 "Notably, the structural model is supported by isothermal titration calorimetry (ITC) experiments (Fig. 2B). In ITC, βarr2 exhibits a biphasic binding pattern with C7pp2, and data fitting to a two-site binding model indicates that C7pp2 contains two distinct binding motifs for βarr2.": Fitting values (KD values) are not shown in the text or in Figure 2B and should be added. KD values are shown in the supplement for other ITC conditions.

***RESPONSE***

We sincerely appreciate the reviewer’s suggestion and agree that including the K_D_ values in Figure 2B will improve clarity and ensure consistency with the ITC data presented in the supplement. To address this, we have revised Figure 2B to explicitly display the K_D_ values, making them easily accessible to readers.

Fig 2B. ITC analysis of the binding of C7pp2 to βarr2. Purified βarr2 proteins were incubated with increasing concentrations of C7pp2 peptide, and the binding parameters were calculated based on the dose-response curve. βarr2 induces biphasic binding by C7pp2. The indicated *K*_D1_ and *K*_D2_ values correspond to site I and site II, respectively.

[Reviewer #2- minor point 4]

Figure 3A - Side chains should match the corresponding ribbon colors as in Figure 2.

***RESPONSE***

We appreciate the reviewer’s suggestion, and as recommended, we have modified the color of the side chains in all structural figures throughout the manuscript to match those of the ribbons. Once again, we sincerely thank the reviewer for providing valuable comments and insightful concerns, and for giving us with the opportunity to address these points and further improve our manuscript.

**Reviewer #3 (Remarks to the Author):**

This manuscript by Ji and coworkers describes a combination of molecular dynamics simulations (MD), biochemical and cell-based experiments to elucidate the activation mechanism of β-arrestin.

Arrestin activation occurs by the binding of the phosphorylated tail or intracellular loops of G protein-coupled receptors (GPCRs). The structures of β-arrestins (also called arrestin2 and arrestin3) in complex with phosphorylated GPCRs or respective phosphopeptides have been elucidated and show that the phosphopeptide forms an intramolecular beta-sheet with arrestin strand beta-1. The peptide thereby replaces arrestin beta-strand 20, which in (inactive) apo arrestin forms an intramolecular beta-sheet with strand beta-1. As a consequence, beta-20 and the entire C-terminus are released from the arrestin core, the arrestin N- and C-domains rotate slightly against each other and the arrestin loop conformations change.

While this activated state is known, it is unclear whether the binding of the peptide occurs via intermediate steps. To elucidate such intermediate steps, the authors use mostly a shortened version of a phosphopeptide derived from the carboxyl terminus of CXCR7, C7pp2, to study the interactions with βarr2. C7pp lacks the last three residues STK of the C-tail, two of which (ST) can also be phosphorylated. A second peptide C7pp3 comprises these residues. The authors carry out MD simulations as well as biochemical and cellular experiments, from which they claim that the C7pp2 peptide displaces as a first step R394 while still keeping the beta-20.beta-1 sheet intact. This would then be an intermediate step during arrestin activation.

However, as indicated below, this evidence is not solid and the reasoning is flawed. For this reason, I cannot recommend this manuscript for publication.

***RESPONSE***

We sincerely appreciate the reviewer’s time and effort in carefully reviewing our manuscript and providing detailed feedback. We acknowledge the importance of the concerns raised and we fully agree that these points are critical and require thorough consideration.

To address the reviewer’s concerns regarding the robustness of our evidence and potential flaws in our reasoning, we have incorporated additional analyses and clarifications to strengthen our study. Additionally, we have carefully revised sections where ambiguity may have led to misinterpretation, ensuring that our conclusions are presented with greater clarity and transparency. In this response, the revisions made in accordance with the reviewer's suggestions have been highlighted in blue text. We hope that our responses are clear, concise, and satisfactory to the reviewer.

**Main points**

[Reviewer #3- main point 1]

MD simulations: the MD simulations were carried out for sets of 7 x 300 ns or 5 x 200 ns. This is much too short to follow a beta-strand release or replacement from a beta-sheet. Typical opening times of individual beta-hairpins are on the order of at least 10 microseconds. The authors give no evidence about the convergence of their simulations. Thus, it is unclear what to conclude much from any of the MD results.

***RESPONSE***

We would like to thank the reviewer for highlighting this issue. As the reviewer very correctly comments, observing the displacement of the βarr2 C-tail in the proposed simulation timescales would be very challenging (due to strong interactions of the inactive C-tail with the βarr2 polar core, as well as due to hydrophobic interaction stabilizing the β-sheet). Because of this, to capture an intermediate state of the displaced C-tail, we simulated spontaneous C-tail inactivation after manual displacement. Since this process is entropy-driven and does not require additional energy input, it can be observed within the sampled timescales (S1 Fig and Authors’ response Fig. 1). Our rationale was that mapping the transition pathways of the C-tail during inactivation would reveal metastable conformations relevant for the activation/inactivation pathway of the C-tail, thereby providing insights into the initial states of C-tail displacement. Building on these data, we developed a computational model of the intermediate βarr2-C7pp2 state, which we subsequently validated through *in vitro* and biophysical assays.

We acknowledge that the presented version of the figure is misleading (i.e. in Fig 1C. the arrow was placed to denote the manual displacement induced in the inactive structure, however it could erroneously suggest the displacement is something we see in the simulations). We have improved the figures and captions, to ensure our simulation protocol is more understandable.

Fig 1C. Ribbon diagram of inactive βarr2. The C-tail of inactive βarr2 is highlighted in red. The polar core region, gate loop, and βXX motif are indicated. A zoomed-in view of the polar core region (right panel) shows an initial step of the simulation. The C-tail of βarr2 (residues 385 to 397) was manually displaced (shown in green) from the initial inactive conformation. The observed spontaneous inactivation of the C-tail during the simulation is indicated by a dotted arrow.

Furthermore, to strengthen our findings we have substantially extended the number of simulation replicates: for spontaneous C-tail inactivation, from 7 × 200 ns to 35 × 200 ns (totaling 9.8 μs instead of 1.4 μs), and for refinement of the docked pose, from 7 × 300 ns to 35 × 300 ns (14.7 μs instead of 2.1 μs) (Fig 1D and 1F). Importantly, an analysis of the obtained data demonstrates that in several replicates we successfully observed the full inactivation of the C-tail (Authors’ response Fig 1). Since the inactive-like conformation of βarr2 represents its native, low-energy conformation, we argue that energetic convergence was achieved in several replicates. Moreover, increasing the number of replicates did not change the conformational equilibrium of the main states observed in our simulations.

**Authors’ response Fig 1.** Analysis of the R394 – D299 distance in the obtained (35 x 200 ns) data set. By analyzing the distance between R394 and D299 we can appreciate that both residues frequently assume a conformation (characterized by a distance equal or lower of 4 Å) characteristic for the inactive conformation of βarr2. This figure replaced to the **S1 Fig**.

Importantly, clustering analysis of the expanded dataset (using the exact same structural cutoffs) remains consistent with our previous findings – both the population and conformational ensemble in the main cluster are highly similar (Authors’ response Fig 2). Overall, we examined the conformation and population of the main R394 cluster in two computational experiments conducted in this study: spontaneous inactivation and docking pose optimization. Specifically, we compared the most populated clusters between the original dataset (7 replicates) and the expanded dataset (35 replicates). Notably we observe that within the expanded dataset, the general shape and population of the most populated cluster remain essentially unchanged. This figure has now been included in the main manuscript (S2 Fig).

**Authors’ response Fig 2.** The main conformational cluster explored by R394 in computational experiments within the study. This figure is now included in the main manuscript as **S2 Fig**.

To further support the validity of our model, we performed unbiased molecular dynamics simulations of the βarr2_IM_-C7pp2 complex (Fig 2A). To assess the convergence of the model, we have now included an additional figure analyzing the stability of βarr2_IM_-C7pp2 interactions. This figure is presented in the main manuscript as S5 Fig. Additionally, to clarify our observation on the model convergence, we have added the following sentence in the manuscript.

Lines 173-174:

“Despite their highly dynamic nature, the observed interactions appear to primarily converge over the course of the simulations in the studied replicates (S5 Fig).”

**S5 Fig. Stability of polar interactions between C7pp2 and βarr2.** (A and B) The distance between residues in site I (pS350 and pT352 of C7pp2; R77, R148, and R166 of βarr2) and site II (E358 of C7pp2 and R394 of βarr2) are monitored during the whole simulation run (panel A, 5 × 300 ns) and the last 50 ns of each simulation replicate (panel B, 5 × 50 ns). For site I, the distance between the phosphorus (P) atom of the phosphorylated residues (pS350 and pT352) and the carbon atom in the guanidinium group (Cζ) of the corresponding arginine residues (R77, R148, and R166) are plotted. For site II, the distance between the Cδ atom of E358 and the Cζ atom of R394 is plotted.

Additionally, we observed distinct convergence patterns between interactions within binding sites I and II (S5 Fig). In binding site II, the interaction between E358 and R394 displays dynamic association and dissociation, accompanied by consistent fluctuations in distance. Notably, one replicate (900–1200 ns in panel A; 150–200 ns in panel B) explores a distinct conformational space, potentially corresponding to a sparsely populated alternative state. This deviation may reflect an escape from a local minimum, capturing a rare but structurally plausible conformation. The dynamic behavior of site II is consistent with an intermediate state expected for this system. In contrast, interactions within binding site I show convergence across most replicates, with the outlier still maintaining stable contacts. These observations support local convergence with occasional transitions.

[Reviewer #3- main point 2]

ITC experiments: Fig. 2B shows a biphasic ITC profile. The horizontal axis is not labeled. I assume it is the molar ratio. No stoichiometry is indicated. The biphasic behavior is used as evidence for a two-site (step-wise) binding mechanism. However, this does not make sense. If the horizontal axis is indeed the molar ratio, then the reaction would give two C7pp2 peptides bound to one βarr2 molecule. How can this be? A GPCR has only one tail. The experiment also lacks a description how the fit curve was obtained. Such a biphasic fit must involve a 2:1 chemical reaction.

***RESPONSE***

We sincerely appreciate the reviewer’s insightful comment and the careful evaluation of our ITC experiments. We acknowledge the importance of accurately interpreting the biphasic ITC profile and ensuring that our conclusions align with physiologically relevant GPCR-arrestin interactions.

As the reviewer pointed out, the biphasic ITC profile may suggest a 2:1 binding model, deviating from the conventional 1:1 GPCR–arrestin interaction. However, we believe that our ITC data reflect the affinities of two distinct binding sites—sites I and II. This biphasic pattern likely results from the experimental design, which involved only the C-terminal peptide of CXCR7 rather than the full-length receptor. Below, we outline the rationale behind our interpretation of the ITC data (Authors’ response Fig 4).

1. Before titration begins, the ITC cell contains only βarr2 (Authors’ response Fig 4A).
2. As C7pp2 is injected, it begins to occupy the higher-affinity site (site I) on βarr2 (Authors’ response Fig 4B).
3. With continued titration and increasing C7pp2 concentration, the lower-affinity site (site II) also begins to be saturated (Authors’ response Fig 4C). Importantly, C7pp2 binding at site II can occur in two different ways:

① **Intramolecular binding:** A C7pp2 peptide that is already bound at site I also interacts with site II, forming an intramolecular 1:1 interaction within a single βarr2 molecule.

② **Intermolecular binding:** A free C7pp2 peptide binds to Site II of a separate βarr2 molecule, resulting in a 1:2 stoichiometry (i.e., two C7pp2 molecules bound to a single βarr2), which may not reflect the physiological situation in cells.

4. As the concentration of free C7pp2 increases, it competes with the bound C7pp2 due to its higher relative abundance, leading to the binding of one or two C7pp2 peptides to βarr2 (Authors’ response Fig 4D).

**Authors’ response Fig 4.** Suggested model for βarr2 and C7pp2 binding observed in ITC. The upper panel illustrates βarr2 and C7pp2 binding interactions. Lower panel displays corresponding molar ratio points, with red arrows indicating key binding events. (A) Initial state of βarr2 in the ITC cell. βarr2 in its inactive state with the C-tail highlighted in green. The potential binding sites for C7pp2, Site I and Site II, are marked with dotted circles. (B) High-affinity binding at Site I. At the start of C7pp2 titration, Site I, the high-affinity binding site, is the first to be occupied by C7pp2. (C) Competitive binding at Site II. As titration progresses and C7pp2 concentration increases, Site II, a lower-affinity binding site, becomes occupied through competitive binding. (D) Saturation state in the ITC cell. βarr2 is fully saturated with C7pp2, forming 1:2 binding complex.

In this ITC binding model, βarr2 and C7pp2 can ultimately form complexes at ratios of either 1:1 or 1:2, consistent with our ITC fitting results, where the inflection point indicating saturation is located between molar ratios of 1.0 and 2.0. This model also aligns with our mutational assay results (S6 Fig), as removing one of the binding sites eliminates the biphasic behavior while simultaneously resulting in a binding ratio approaching 1:1. We believe that this explanation sufficiently accounts for the observed stoichiometry deviation while remaining consistent with our experimental findings.

To increase the readability of this explanation, we revised the corresponding parts as below.

Lines 182-188:

“Notably, the structural model is supported by isothermal titration calorimetry (ITC) experiments (Fig 2B). In these experiments, βarr2 exhibits a biphasic binding profile with C7pp2, and fitting the data to a two-site binding model suggests that one or two C7pp2 peptides can bind to βarr2. This non-physiological 1:2 stoichiometry (receptor:βarr2 = 1:1 in cells) likely arises from the use of the isolated C-terminal peptide of CXCR7 rather than the full-length receptor. Nonetheless, the biphasic behavior clearly indicates that C7pp2 harbors two distinct binding motifs for βarr2—one that interacts at lower concentrations and another that becomes engaged only at higher concentrations during the titration.”

[Reviewer #3- main point 3]

Tango assay (Fig. 3 C-E): the assay shows that CXCR7-E354 is important for βarr2 recruitment, but not more. I.e. it does not give evidence that the claimed intermediate state exists.

***RESPONSE***

We appreciate the reviewer’s insightful comment. As noted, the mutational data do not directly support the presence of an intermediate state in cells. Since such a state is likely transient and short-lived, validating its existence in a cellular context is inherently challenging. Nonetheless, we believe that the E358A mutant indirectly supports the functional importance of E358, which we propose plays a critical role in destabilizing the polar core during βarr2 activation.

To address this point more clearly, we have added a more detailed description of the confocal microscopy and Tango assay data as below. We also clarify that while the observed loss of function does not definitively confirm the existence of an intermediate state, it does provide indirect support for the role of E358 in the activation process of βarr2.

Accordingly, we have revised the manuscript to include additional explanations in both the figure legend and the main text to ensure that readers understand the rationale for performing the Tango assay and interpret its results appropriately.

Lines 262-269:

“Moreover, confocal microscopy revealed that, unlike CXCR7^WT^, the CXCR7^E358A^ mutant failed to induce βarr2 redistribution from the plasma membrane to intracellular endosomes upon CXCL12 stimulation, suggesting impaired receptor internalization (Fig 3C). Additionally, the Tango assay demonstrated that CXCR7^E358A^ exhibited a significant reduction in βarr2 recruitment in response to CXCL12, confirming that the E358A mutation disrupts βarr2 engagement and downstream trafficking (Fig 3E). While the observed loss of function does not definitively confirm the existence of an intermediate state, it does provide indirect support for the role of E358 in the activation process of βarr2.”

Lines 226-231:

“**Fig 3C.** CXCL12-induced trafficking of βarr2 as monitored using confocal microscopy in HEK293 cells expressing CXCR7^WT^ and CXCR7^E358A^. The "Surface" images depict βarr2 localization at the plasma membrane shortly after agonist stimulation, representing initial recruitment of βarr2 to CXCR7. The "Endosomal" images show intracellular βarr2 localization at later time points, indicating receptor internalization. Scale bar is 10 μm.”

[Reviewer #3- main point 4-1]

HDX experiments (Fig. 4): The observed differences between C7pp2-bound and -free βarr2 are in general very small. The claimed significant differences for decreased exchange (blue asterisks) are particularly small and are observed only for the first time point, which may be an experimental variation after mixing.

***RESPONSE***

We appreciate the reviewer’s concern regarding the small differences observed, particularly at the first time point. To clarify, the βarr2 alone and βarr2 with C7pp2 samples were diluted into the D_2_O buffer in an identical manner, and the observed differences at the first time point are not attributable to mixing effects. Rather, these differences likely arise from distinct conformations of βarr2 in the presence and absence of C7pp2. It is also important to note that the time points are measured after D_2_O incubation, not after peptide mixing.

While the observed differences are small, they reflect subtle conformational changes induced by C7pp2 co-incubation. Comparatively, in our recently published study, co-incubation with V_2_Rpp, the phosphorylated C-terminal tail of Vasopressin receptor type 2, resulted in broader HDX changes and larger differences [11]. Although direct comparisons between the two studies should be made cautiously due to differences in experimental setups, we observed increased HDX at the βarr2 C-terminal tail, although to a lesser extent compared to the V_2_Rpp-bound state. This result supports the idea that the binding of C7pp2 induces conformational changes through interactions with the βarr2 C-tail, even if it does not fully displace the C-tail as observed in the V_2_Rpp-bound state.

Finally, while the differences appear small in the graphs, the differences were consistently greater than 0.2 Da (see S1 Dataset), supporting their reliability.

[Reviewer #3- main point 4-2]

More importantly as indicated in the methods 2 μL of protein samples were mixed with 28 μL of D2O buffer. So, the final deuterium uptake should be 1-2/28 = 93%. However, the uptake is maximally 60% and varies strongly between the detected stretches albeit saturation seems to be achieved. This is completely unclear.

***RESPONSE***

We thank the reviewer for pointing out this important detail. While the theoretical deuterium uptake can reach approximately 98%, several factors can influence the final observed uptake:

1. **Protein conformation:** The accessibility of amide hydrogens to the D_2_O buffer depends on the protein's conformation. Regions where amide hydrogens are buried or participate in stable hydrogen bonds exhibit lower deuterium uptake, even under extended exchange conditions.
2. **Back-exchange:** After HDX, samples are mixed with a quench buffer at a 1:1 ratio. As the quench buffer contains H_2_O, the deuterium incorporated into the protein partially exchanges back to hydrogen, reducing the observed deuterium uptake. This phenomenon is well-documented and described in the literature [12]. Back-exchange correction is typically applied when determining absolute deuteration levels. However, for comparative studies such as ours (βarr2 alone vs. βarr2 with C7pp2), back-exchange correction is unnecessary as relative differences are unaffected.

As the reviewer pointed out, full deuterium uptake can be up to 98%. There are other factors that affect the final deuterium uptake. Moreover, based on our extensive experience with HDX-MS analyses of arrestins [2–11], we have observed that achieving fully deuterated samples for arrestins often leads to aggregation, which complicates or even prevents back-exchange correction. We hope this explanation clarifies the observed uptake levels and the experimental limitations inherent to HDX-MS.

[Reviewer #3- main point 4-3]

As a small additional point, it is also unclear what the following sentence in the figure legend means 'Differences smaller than 0.2 Da were not considered significant.'

***RESPONSE***

This statement is included in accordance with the HDX-MS reporting standards outlined in the literature [12]. To avoid misinterpretations caused by noise in the mass spectra, only deuterium uptake differences exceeding 0.2 Da were considered significant. This threshold was established after careful examination of all mass spectra across the analyzed samples to ensure the reliability of the observed differences. Thus, any uptake differences below 0.2 Da were likely attributable to noise and were excluded from interpretation.

**References**

1.	Sarma P, Carino CMC, Seetharama D, Pandey S, Dwivedi-Agnihotri H, Rui X, et al. Molecular insights into intrinsic transducer-coupling bias in the CXCR4-CXCR7 system. Nat Commun. 2023;14(1):4808.

2.	Yun Y, Kim DK, Seo M-D, Kim K-M, Chung KY. Different conformational dynamics of β-arrestin1 and β-arrestin2 analyzed by hydrogen/deuterium exchange mass spectrometry. Biochem Biophys Res Commun. 2015;457(1):50-7.

3.	Kim DK, Yun Y, Kim HR, Seo M-D, Chung KY. Different conformational dynamics of various active states of β-arrestin1 analyzed by hydrogen/deuterium exchange mass spectrometry. J Struct Biol. 2015;190(2):250-9.

4.	Park JY, Qu C-x, Li R-r, Yang F, Yu X, Tian Z-m, et al. Structural mechanism of the arrestin-3/JNK3 interaction. Structure. 2019;27(7):1162-70. e3.

5.	Kang H, Yang H-S, Ki AY, Ko S-B, Kim KW, Shim CY, et al. Conformational dynamics and functional implications of phosphorylated β-arrestins. Structure. 2020;28(3):314-23. e3.

6.	Min K, Yoon H-J, Park JY, Baidya M, Dwivedi-Agnihotri H, Maharana J, et al. Crystal structure of β-arrestin 2 in complex with CXCR7 phosphopeptide. Structure. 2020;28(9):1014-23. e4.

7.	Qu C, Park JY, Yun MW, He Q-t, Yang F, Kim K, et al. Scaffolding mechanism of arrestin-2 in the cRaf/MEK1/ERK signaling cascade. Proc Natl Acad Sci U S A. 2021;118(37):e2026491118.

8.	Yun Y, Yoon H-J, Jeong Y, Choi Y, Jang S, Chung KY, et al. GPCR targeting of E3 ubiquitin ligase MDM2 by inactive β-arrestin. Proc Natl Acad Sci U S A. 2023;120(28):e2301934120.

9.	Maharana J, Sano FK, Sarma P, Yadav MK, Duan L, Stepniewski TM, et al. Molecular insights into atypical modes of β-arrestin interaction with seven transmembrane receptors. Science. 2024;383(6678):101-8.

10.	Kim K, Ashim J, Ham D, Yu W, Chung KY. Roles of the gate loop in β-arrestin-1 conformational dynamics and phosphorylated receptor interaction. Structure. 2024.

11.	Kim K, Chung KY. Molecular mechanism of β-arrestin-2 pre-activation by phosphatidylinositol 4, 5-bisphosphate. EMBO Rep. 2024;25(10):4190-205.

12.	Masson GR, Burke JE, Ahn NG, Anand GS, Borchers C, Brier S, et al. Recommendations for performing, interpreting and reporting hydrogen deuterium exchange mass spectrometry (HDX-MS) experiments. Nat Methods. 2019;16(7):595-602.
